# Lectures on the Diseases of Women

**Published:** 1858-04

**Authors:** 


ART. V.—*Lectures on the Diseases of Women.* By CHARLES WEST, M. D., Fellow of the Royal College of Physicians; Examiner in Midwifery, at the Royal College of Surgeons of England, &c., &c., &c. Part I, Diseases of the Uterus. Philadelphia: Blanchard & Lea. 1857.

This work of Dr. West is doubtless well known by this time to a great proportion of the profession, as it has been given to them in monthly instalments in the pages of the *Medical Library and News*, and now being completed in that serial, is given to the public in a neat volume of about three hundred and twenty pages.

The author is too well and too favorably known through his work on Diseases of Children, to stand in need of especial commendation of anything which may subsequently emanate from his pen. He enters earnestly into his subject, and arrests the attention of his reader by the perspicuity of his style, and the rapidity with which he pursues his subject and cites his authorities.

Dr. West belongs to that portion of the profession, who if they do not ignore the use of the speculum in the diagnosis of uterine diseases, depreciate its value and importance by denying the frequency of the existence of local lesions, and their influence in developing that train of constitutional disturbances which go to make up a large proportion of that long catalogue of ills, which find their places in the list of Female Diseases. These opinions give a manifest bias to all his views, and their influence may be traced in his teachings.

The volume before us, unlike his *Croonian Lectures on Ulceration of the Os Uteri*, is too general in its character, to allow the partizan character, into which the discussion of this latter subject has betrayed the disputants, to interfere with its real and practical value. His skepticism upon this one subject, finds bounds when he enters upon the discussion of those subjects which from their nature preclude a reference to mooted points in pathology, and furnish no opportunity for the championship of a party.

A work of such general character furnishes, too, fewer salient points for criticism than one which comes avowedly forth as the exponent of some peculiar doctrine. Its teachings find more ready acceptance, and they are admitted at once to a place in the mind of the practitioner, and soon put to the test of every-day practice.

The volume opens with two sensible and practical chapters on the importance of accuracy in diagnosis; a notice of some of the peculiar elements characterizing the female system, which impress upon the diseases incident to the sex their especial modifications and forms; the manner of making tactile examination of the abdomen, and of the generative organs; and of the method of employing the uterine sound and the speculum.

Among the illustrative cases of errors in diagnosis, the following example, in these days when such frequent recourse is had to examinations of the urine, and such serious results are predicated, upon the appearance of albumen in the renal secretions, will bear quoting:

"A middle-aged woman complained of frequent desire to pass water, and of discomfort in voiding it; she was dyspeptic, and out of health. Her urine was tested, and found to contain albumen; and the irritable state of her bladder was assumed to be dependent on the disease of her kidneys. Treatment improved her general health, but brought no relief to her dysuria. At length careful observation discovered the albumen to be due to the admixture of vaginal discharges with her urine: a not infrequent source of it in women who suffer from leucorrhœa, while examination, which had been delayed too long, detected a small vascular tumor just within the orifice of the urethra, to the irritation produced by which her symptoms were due, as was shown by their immediate disappearance on its removal."

His directions for the manner of using the uterine sound are simply what the manipulation requires, and being confined to the mode of introducing it, does not admit of any criticism. He gives at length the manner of employing the speculum, but while faithfully teaching the methods of using it, manages to envelop his instructions in a cloud of doubts of its utility, or the necessity of its employment, except upon extraordinary occasions. We quote directly our author, and allow him the use of his own language: "To answer the broad question, 'What is your opinion of the speculum?' I feel, on the other hand, to be a very difficult matter, and to expose me to much risk of being misunderstood. I will, however, do my best to reply to the inquiry. Those who first introduced the speculum into practice, employed it for two purposes; partly as furnishing a new means of diagnosis, partly as enabling them to adopt various modes of local treatment, which without it, were impracticable. Now I believe that the advantages of these topical medications for which the speculum is needed, has been greatly overrated; though there are some cases, and those, such as have proved most rebellious under other plans of treatment, in which these local measures may be resorted to with the most signal advantage. In estimating the value of the speculum as a means of diagnosis, I think that the advances in knowledge of uterine diseases, of which it was the indirect occasion by the impulse which it gave to their study, are sometimes confounded with those positive additions to our information, which we owe exclusively to the use of that instrument."

Any admissions of the value of the instrument as a means of diagnosis, demanded by truth, are apparently reluctantly expressed, and uttered with great caution. At the same time, he indulges in no violence of language, and decries the employment of intemperate opposition, and the imputation of dishonest motives to the advocates of the instrument.

He makes a practical suggestion as to the insufficient length of many of the instruments in use, and of the failure from this cause, to reach the os uteri; and agrees with the opinion of the late Professor Lisfranc, that a speculum ought to be at least seven inches long.

Menstruation, and its Disorders, are discussed in the third, fourth and fifth lectures. Tardy puberty, and amenorrhœa from malformations are first considered, when he proceeds to the consideration of *amenorrhœa from causes which require the interference of the physician.*

There are two conditions of the system under which this derangement of the health occurs, one of *plethora*, in the other of *anæmia;* but with a tendency of the former to pass into the latter, a transition which often takes place very speedily.

These conditions, with their accompanying symptoms, and appropriate modes of treatment, are graphically described, but we do not find anything especially new in the description, to add to our stock of knowledge in the pathology or treatment of the disease.

*Menorrhagia* is next considered, under a classification which he deems the only one of real practical utility; viz., 1st. Depending on some cause seated in the constitution generally. 2d. Depending on some affection of the sexual system.

At this point Dr. West says: "One caution is, perhaps, worth giving before I say anything more about menorrhagia. It is, that every excessive hemorrhage from the unimpregnated uterus, during the years of sexual activity, is not necessarily menorrhagia. Women themselves are apt so to regard all losses of blood during that period of their life, and practitioners are too often guilty of the same oversight. Menorrhagia is an excess of menstrual discharge, an overabundant hemorrhage, the cause of which, in the first instance, is that congestion of the sexual organs which attends maturation and escape of an ovule from the ovary. Outbursts of bleeding may take place from the womb in some cases where the menses have been long suppressed, affording relief to the system, or even by their excess jeopardizing the patient's well being, and this with no more real reference to the function of which menstruation is the sign, than exists in a case of hemorrage from the bowels, or of bleeding from hemorrhoids. In the same way, too, a patient may bleed to death from a cancer of the womb, or from a polypus, or fibrous tumor of that organ, and yet such hemorrhage may be no real menorrhagia."

But we have not space or time to devote to the consideration of the several pages which he gives to this subject, including treatment, and the indications for the several modifications of the same.

The next section is devoted to the consideration of Dysmenorrhœa. But we do not discover in it anything calling for especial notice. The symptoms accompanying the several forms of painful menstruation are carefully given, and the treatment applicable to each variety indicated with equal painstaking. The remaining fourteen lectures or sections of the book are occupied with diseases especially affecting the uterus.

In this connection we may add, what we have omitted before to notice, that notwithstanding the fullness of the title, the author does not profess this volume to be a complete treatise upon female diseases, but designs to continue the subject in another volume, to be published in a year or two, in which will be considered the diseases of the ovaries, and of all the parts connected with the uterus; of the bladder, vagina, and external organs.

In the volume before us, he considers inflammation of the uterus, acute and chronic hypertrophy, ulceration of the os uteri, cervical leucorrhœa, displacements of the uterus, uterine tumors and outgrowths, and cancer of the uterus. A series of subjects sufficiently ample to be far beyond our power to do full justice in the compass of a review proportioned to the number of our pages. We shall skim but lightly over the several chapters, stopping only sufficiently long to indicate the character of their contents, and shall by no means either duplicate the volume, or render by our abstracts, reference to it unnecessary.

By way of introduction to the diseases of the uterus, and particularly of inflammations, our author refers to some of the conditions which follow delivery, especially to its enlarged state from want of due exercise of the process of involution of the organ, by which it remains permanently, or until relieved by treatment, in a state of congestion. Cases of a more genuine hypertrophy of the womb, he says, take place independently of pregnancy, which he charges to a condition of uterine congestion, induced by the imperfect, or incomplete act of marital intercourse, and intimates that in such cases, an enquiry into the condition of the virile powers of the opposite sex is not beneath the notice of the physician in the course of his treatment.

One other form of simple uterine hypertrophy he notices in this connection, in which the enlargement is limited to the neck of the uterus. It is usually consequent on child-bearing, and is perhaps, more strictly speaking, rather the result of a partial deficiency of involution of the uterus than the effects of a genuine hypertrophy of the part. He has, however, met with it in sterile women, and once even in an unmarried girl. The chief increase is in length, the portio vaginalis, instead of being half or three quarters of an inch in length, measuring an inch and a half or two, or even three inches. The only treatment is removal, an operation which he by no means recommends.

*Acute inflammation* of the unimpregnated uterus is an ailment universally admitted to be of rare occurrence. It may, however, be caused by the extension of gonorrhœal inflammation; sudden suppression of the menses; and by unaccustomed and intemperate sexual intercourse. In a fatal case of acute uterine inflammation, accompanied by peritonitis, a fibrous tumor of the size of a hen's egg was found imbedded in the posterior wall of the uterus.

A full discussion of the points involved in the above subjects brings our author to the consideration of *chronic inflammations* of the uterus, which he treats in the two succeeding sections.

This furnishes him with the first legitimate opportunity he has had, to indulge his love for discussion, and enunciate the views of his school upon the subject of the influence of local uterine disease, and of the pathological non-importance of ulceration of the os uteri.

The section is a recapitulation of the arguments employed to disprove the position assumed by those who discover, if not in one the cause of the other, at least so close a connection between the local disease and the attendant constitutional disturbances, that the treatment of the former is essential to ensure a mitigation of the latter symptoms. Reference is made to his Croonian Lectures for a greater amplification of the subject.

We can not but regard the manner in which Dr. West endeavors to dispose of the question which most unavoidably, and we think somewhat embarrassingly, meets him in the way, how to account for the repeated cures attendant upon local medication,—as a most "weak invention." Committed to one side of the argument, like disputants in a debate, he is compelled to meet all the advances of his opponents, by a reply of some sort, even if sound logic is sacrificed in the discussion. The patients benefited and cured by local treatment after years of suffering, and ineffectual general treatment, will, however, continue to appear to confound these arguments, and furnish the most convincing proof of the strength of the position of those he opposes.

He admits, however, that pregnancy, delivery, or miscarriage may develop ailments which point to a purely local cause for their origin. But even in these cases, he attributes the symptoms to uterine congestion following upon the conditions of pregnancy and delivery, and the cases he cites are given to negative, as far as possible, the theory of their dependence upon ulceration or abrasion, or any form of disease which may be specifically located at the os, or within the cervix.

There is one gratifying feature in the manner in which Dr. West has treated the subject in this connection. He bases his objections upon purely pathological grounds. He employs none of the puerile arguments, which of late have been so freely used, ringing the changes upon outraged female modesty. That pseudo knight-errantry in behalf of the virtue and modesty of the sex, avowed by some, has found no champion in these pages. He has indulged in no declarations, such as some of those enlisted in this crusade for the preservation of the honor of women, have employed, and who have avowed, that unless some check was placed upon the use of the speculum, they should feel compelled to abandon the treatment of female diseases!*

We shall make no attempt to analyze the five sections, including seventy pages, devoted to the consideration of displacements of the uterus. This is the less necessary, as fewer considerations of pathological importance are concerned in the production and maintenance of the malpositions of the uterus and the vaginal walls, than in the other morbid conditions of the sexual

* See debate reported in the American Journal of the Medical Sciences, October, 1850, p.p. 533,–42.

system discussed in the volume. These displacements generally, although not invariably, take place in consequence of pregnancy, and delivery at full term, or the anticipation of that event by miscarriage. The attendant symptoms are those which result principally from the abnormal relations of the parts, and the consequent distress from the malpositions of the organs and their connections involved.

The chief interest in the subject is centered in the means of diagnosis of the different degrees of misplacement, and the adaptation of proper methods of treatment. Mechanical means of support, except in the slighter cases, are requisite for the relief of the patient, and the choice of the proper instrument constitutes the prominent indication in the treatment.

These several subjects are all treated in an eminently practical manner, and the instructions are sound.

The consideration of the subjects of Uterine Tumors and Outgrowths occupies four lectures, or chapters. The first of these, the fourteenth lecture, is devoted to the several forms of polypi.

Of the minute description which he gives of uterine polypus, through its various forms of organization and development, from the simple *mucous polypus*, we content ourselves with extracting the following passage only, relative to the means of making a diagnosis in these cases:

"I do not know of any special difficulty attending the *diagnosis* of these outgrowths, nor of any particular rules which can be laid down for the avoidance of error. The very small polypi are sometimes scarcely perceptible by the finger, and I have already referred to the enlargement of the cervix which they occasionally produce, and which is likely to mislead the unwary. The only rule that can be given for practical guidance is, however, this: that in no case of long-continued menorrhagia should we be content with mere digital examination, but should invariably employ the speculum; and further, if no satisfactory conclusion be thereby arrived at, we should dilate the os uteri with sponge tents, in order that the cervical canal may be brought within reach both of examination with the finger and with the speculum. If these precautions be neglected, the patient whom we have failed to relieve, may place herself under some more careful practitioner, who will at once detect the cause of her symptoms, and cure her by an extremely simple operation."

In removing "*these small outgrowths*," he recommends always the use of the bivalve speculum, in employing for that purpose either the forceps or scissors:

"To attempt their removal by means of forceps or scissors, simply guided by the hand, is at best but a bungling mode of proceeding, while, besides, the risk of hemorrhage is much greater than it would be if, after the removal of the polypus, the part whence it sprang were touched with the solid nitrate of silver, a precaution which I now never omit."

As a matter of prudence, he deems it necessary that the patient should be kept in bed a day or two after the operation.

The fifteenth, sixteenth, and part of the seventeenth lectures are devoted to the consideration of fibrous tumors of the uterus.

After a concise description of the peculiar anatomical characteristics of these tumors, and of the several different situations from which these growths may proceed, he passes on to a notice of their size and number, the changes which they undergo while in the uterine tissues, the means which nature at times seems to adopt for the purpose of getting rid of their presence; and a consideration as full as statistics can furnish data, of the influence of age on the production of these morbid growths. The symptoms attendant upon their development, are minutely given; and the means of establishing a diagnosis in contra-distinction to ovarian tumor, from flexions of the uterus, uterine cancer, and pregnancy, are carefully considered.

The several subjects are treated in an eminently practical manner, and we think the physician can scarcely arise from the perusal of the several pages devoted to their consideration, without a consciousness of being fully compensated for the time devoted to their study.

While admitting the hopeless task of treating an irremediable disease, he deprecates the abandoning entirely the patient; but urges a palliative treatment, to husband the strength, and to retard the growth of the tumors. The disease often has pauses in its course; in some rare cases a perfect recovery is effected by the hands of nature; and their development is generally slow, and much less rapidly fatal than many other forms of uterine disease.

He has little faith in medication to produce their absorption. Mercurial preparations have no such influence; and the alleged powers of iodine seem to have been much over-rated. He thinks, however, that the rapid increase of these growths is sometimes restrained by this agent, and is therefore accustomed to employ it. Its use should be continued for many months, and the patient only slowly brought under its influence. The mineral waters of Kreunach, in Germany, which contain both iodine and bromine, have acquired, and apparently with justice, considerable reputation for the special influence which they exert over enlargements and fibrous tumors of the uterus.

Surgical interference he regards with but little favor. Such of them a spring from a distinct pedicle, and hang down into, or beyond the uterine cavity, admit of removal either by the knife or the ligature. He regards the opening of the abdomen for the extirpation of a fibrous tumor, a proceeding to be altogether deprecated. Nor does he regard with more favor the enucleation of these tumors by incisions made through the os uteris, or the lower segment of the womb.

The balance of the chapter he devotes to *Fibrous Polypus*. And for their removal prefers their incision by the knife, to the use of the ligature.

The balance of the volume, three lectures, is devoted to the consideration of malignant, or cancerous diseases of the uterus. But a lack of space, as well as a want of time, compel us to forego an analysis of the contents of the several pages devoted to the subject.

In bringing our notice of this work of Dr. West to a conclusion, we can do no other way, than speak of it in terms of commendation. The practitioner will find it convenient as a work of reference, and if he remember the peculiar tendency of the school or sect to which the author belongs, and make due allowance for the bias of the doctrines which they deem it essential to teach, will find himself repaid for the time devoted to its perusal.

While this volume will not detract any from the reputation of Dr. West, we are compelled to say, that we doubt whether it will do as much towards the building up, or maintaining a reputation, as did his work on Diseases of Children. The *eclat* which accompanied the publication of the latter work may, perhaps, have been unfortunate, inasmuch as it renders the profession unsatisfied, unless by a succession of works equally brilliant. N.

